# Complete Genome Sequence of *Acinetobacter baumannii* Strain B8342, a Motility-Positive Clinical Isolate

**DOI:** 10.1128/genomeA.00925-15

**Published:** 2015-08-20

**Authors:** Saranya Vijaykumar, Veeraraghavan Balaji, Indranil Biswas

**Affiliations:** a Department of Microbiology, Christian Medical College, Vellore, Tamil Nadu, India; b Department of Microbiology, Molecular Genetics and Immunology, University of Kansas Medical Center, Kansas City, Kansas, USA

## Abstract

*Acinetobacter baumannii* is an emerging Gram-negative pathogen responsible for health care–associated infections. In this study, we determined the genome of a motility-positive clinical strain, B8342, isolated from a hospital in southern India. The B8342 genome, which is 3.94 Mbp, was generated by *de novo* assembly of PacBio long-read sequencing data.

## GENOME ANNOUNCEMENT

*Acinetobacter baumannii* is an emerging nosocomial pathogen responsible for health care–associated infections, which have been steadily increasing in recent years. *A. baumannii* can cause a variety of infections, including bacteremia, meningitis, skin and soft tissue infections, ventilator-associated pneumonia, and urinary tract infections (1). Since the pathogen has significant intrinsic resistance to antibiotics and an extraordinary ability to acquire novel resistance genes, the infections are increasingly difficult to control (2). Although the organism is classified as nonmotile, some isolates display twitching and swarming-like motilities (3).

We have isolated an *A. baumannii* strain (B8342) from a bloodstream infection in a 1-year-old female patient admitted to Christian Medical College, Vellore, India. This isolate produces a degree of biofilm similar to that of strain ATCC 19606 and displays a moderate level of both swarming-like and twitching motility. Furthermore, the B8342 strain is susceptible to all the traditional antibiotics.

To understand the molecular basis of motility, we determined the complete genome sequence of *A. baumannii* B8342 using PacBio single-molecule real-time (SMRT) technology (4). A 3- to 20-kb library of the genomic DNA was prepared for P6/C4 chemistry without BluePippin size selection. The PacBio RSII sequencing platform generated 69,026 reads, with a mean read length of 10,132 bp from one SMRT cell. The reads were assembled *de novo* with the Hierarchical Genome Assembly Process 3 (HGAP3) (5) in SMRTAnalysis version 2.3.0. The best assembly was selected and the circular contig was trimmed with Minimus 2 (6). Base modifications were detected with SMRTAnalysis using default parameters. The assembled B8342 genome consists of a single circular chromosome with 3,947,826 bp containing a 39.1% GC content.

The genome was annotated using Analysis Engine at the University of Maryland (7) and confirmed using BASys (8). The genome predicts 3,789 open reading frames, 18 rRNA genes, one transfer-messenger RNA (tmRNA), and 74 tRNA genes. Five motifs with m6-adenosine methylation were identified in the genome. Ori-Finder mapped the putative replication origin between positions 1,553,230 to 1,553,899 (9). Analysis at the CGE server (http://www.cbs.dtu.dk/services) indicates that B8342 belongs to an unknown sequence type, since it failed to match with any known multilocus sequence types. ResFinder-2.1 analysis at the CGE server returned only the *blaOXA*-106 gene and no other resistance genes in the B8342 genome. Analysis by antiSMASH suggests five putative secondary metabolite gene clusters, including the acinetobactin and acinetoferrin biosynthesis genes (10). PHAST analysis predicted four complete and one incomplete prophage sequences in the genome (11). IslandViewer3 (12) analysis suggests that the B8342 genome contains at least 15 genomic islands. Similarly, ISFinder analysis (13) indicates that the genome contains numerous insertion elements (IS), the majority belonging to the IS*3*, IS*5*, IS*66*, and IS*256* families. Finally, CRISPRFinder analysis (14) suggests that the B8342 genome encodes at least one confirmed and possibly three additional clustered regularly interspaced short palindromic repeat (CRISPR) sequences. The availability of the complete genome sequence of B8342 will be valuable for further comparative genomic analysis, development of molecular diagnostic tools, and understanding molecular mechanisms of motility in this important human pathogen.

### Nucleotide sequence accession numbers.

The *A. baumannii* B8342 genome sequence was deposited in DDBJ/EMBL/GenBank under the accession number LFYZ00000000. The version described in this manuscript is version LFYZ00000000.1.
